# Increased survival and proliferation of the epidemic strain *Mycobacterium abscessus* subsp. *massiliense* CRM0019 in alveolar epithelial cells

**DOI:** 10.1186/s12866-017-1102-7

**Published:** 2017-09-13

**Authors:** Giovanni Monteiro Ribeiro, Cristianne Kayoko Matsumoto, Fernando Real, Daniela Teixeira, Rafael Silva Duarte, Renato Arruda Mortara, Sylvia Cardoso Leão, Cristiane de Souza Carvalho-Wodarz

**Affiliations:** 1 Departamento de Microbiologia, Imunologia e Parasitologia, Escola Paulista de Medicina, Universidade Federal de São Paulo, São Paulo, SP Brazil; 2 Laboratoire Entrée muqueuse du VIH et Immunité muqueuse, Department Infection, Immunité et Inflammation, Institut Cochin, Paris, France; 3 Laboratório de Micobactérias, Instituto de Microbiologia Professor Paulo de Góes, Cidade Universitária, Rio de Janeiro, Brazil; 4 Department of Drug Delivery, Helmholtz Institute for Pharmaceutical Research Saarland (HIPS), Helmholtz Centre for Infection Research (HZI), Saarbrücken, Germany

**Keywords:** *Mycobacterium abscessus*, Phagosome, Acidification, A549, Macrophages, CRM0019

## Abstract

**Background:**

Outbreaks of infections caused by rapidly growing mycobacteria have been reported worldwide generally associated with medical procedures. *Mycobacterium abscessus* subsp. *massiliense* CRM0019 was obtained during an epidemic of postsurgical infections and was characterized by increased persistence in vivo. To better understand the successful survival strategies of this microorganism, we evaluated its infectivity and proliferation in macrophages (RAW and BMDM) and alveolar epithelial cells (A549). For that, we assessed the following parameters, for both *M. abscessus* CRM0019 as well as the reference strain *M. abscessus* ATCC 19977: internalization, intracellular survival for up 3 days, competence to subvert lysosome fusion and the intracellular survival after cell reinfection.

**Results:**

CRM0019 and ATCC 19977 strains showed the same internalization rate (approximately 30% after 6 h infection), in both A549 and RAW cells. However, colony forming units data showed that CRM0019 survived better in A549 cells than the ATCC 19977 strain. Phagosomal characteristics of CRM0019 showed the bacteria inside tight phagosomes in A549 cells, contrasting to the loosely phagosomal membrane in macrophages. This observation holds for the ATCC 19977 strain in both cell types. The competence to subvert lysosome fusion was assessed by acidification and acquisition of lysosomal protein. For *M. abscessus* strains the phagosomes were acidified in all cell lines; nevertheless, the acquisition of lysosomal protein was reduced by CRM0019 compared to the ATCC 19977 strain, in A549 cells. Conversely, in macrophages, both *M. abscessus* strains were located in mature phagosomes, however without bacterial death. Once recovered from macrophages *M. abscessus* could establish a new intracellular infection. Nevertheless, only CRM0019 showed a higher growth rate in A549, increasing nearly 10-fold after 48 and 72 h.

**Conclusion:**

*M. abscessus* CRM0019 creates a protective and replicative niche in alveolar epithelial cells mainly by avoiding phagosome maturation. Once recovered from infected macrophages, CRM0019 remains infective and displays greater intracellular growth in A549 cells compared to the ATCC 19977 strain. This evasion strategy in alveolar epithelial cells may contribute to the long survival of the CRM0019 strain in the host and thus to the inefficacy of in vivo treatment.

**Electronic supplementary material:**

The online version of this article (10.1186/s12866-017-1102-7) contains supplementary material, which is available to authorized users.

## Background

*Mycobacterium abscessus* is a nontuberculous mycobacterium (NTM) widely distributed in the environment. This bacterium is responsible for lung diseases [1, 2] and healthcare-associated extrapulmonary infections [3–5]. *M. abscessus* is indeed the major pulmonary pathogen within the rapid-growing mycobacteria (RGM) group [2], and it has been the most frequent NTM found in the lungs of cystic fibrosis (CF) patients [6–8].

As for other NTM, *M. abscessus* is present in environmental reservoirs (e.g. water and soil) and has been recently isolated from drinking water [9–11]. The acquisition of this bacterium is therefore most likely to occur from the environment, rather than via person-to-person transmission [12]. Despite sharing genes typically found in environmental organisms [13], *M. abscessus* also harbors genes characteristic of pathogenic bacteria [14, 15]. Likewise, it is an intracellular pathogen of macrophages and free-living amoebas [16, 17].

*M. abscessus* has been classified into three subspecies that are officially accepted: *M. abscessus* subsp. *abscessus, M. abscessus* subsp. *massiliense* and *M. abscessus* subsp. *bolletii* [18]. These subspecies cause similar diseases but can be differentiated by PCR-restriction enzyme analysis (PRA) of the *hsp65* gene, *rpoB* gene sequencing and polymorphisms in the *erm* gene [19]. *M. abscessus* pathogenicity is closely related to its colony morphology on an agar plate: organisms without glycopeptidolipids (GPLs) on their surface show a rough (R) colony morphology, while those with GPLs display a smooth (S) morphology [16, 20–22]. The S variant is motile, biofilm-forming, and less virulent [16, 21, 23]. By contrast, the R variant is non-motile, but more virulent than the S variant [16]. In the lung, *M. abscessus* infection appears to be associated with the R variant, which has a highly persistent behavior [16, 24, 25]. Even so, both morphotypes R and S can be isolated from clinical samples [23, 26, 27], and an interchange between these forms may possibly occur [16, 21]. The clinical isolate *M. abscessus* subsp. *massiliense* CRM0019 was obtained during an epidemic of postsurgical infections related to laparoscopic, arthroscopic and plastic surgeries in 2006, in Rio de Janeiro city, Brazil [28–30] and showed a smooth morphotype upon isolation (“personal communication” by Dr. Rafael Duarte). This isolate belongs to a strain that affected more than 2000 patients between 2004 and 2008 [29, 30], and has substantial tolerance to high concentrations of glutaraldehyde [31].

The numerous cases of *M. abscessus* infection related to a single clone reported in Brazil raised questions about the transmission of this bacterium and its survival in the host. From these questions, studies have mostly been conducted regarding bacterial survival. CRM0019 has increased persistence in vivo [32]*,* which can be attributed to the capacity of *M. abscessus* to manipulate host immune responses in macrophages, epithelial cells, endothelial cells and neutrophils [33–38]. Nonetheless, little is known about the interaction of the CRM0019 isolate with lysosomes or its capacity for reinfection. This is an essential question to address in order to understand the successful intracellular survival of pathogenic mycobacteria, classically represented by *M. tuberculosis*, which arrests progression of phagosome maturation [39, 40]. Macrophages often fail to kill pathogenic mycobacteria, resulting in survival and proliferation of the bacilli [41–43] as observed for other NTM [44, 45] including the CRM0019 isolate [32].

Besides macrophages, the respiratory epithelium plays an important role in defending against respiratory pathogens [46]. There is evidence that epithelial cells can act as a replicative niche for *Mycobacterium* [47–52]. Even so, their contribution to the pathogenicity in humans is not as yet clear. A recent study using a human ex vivo lung tissue culture model reinforced the evidence for mycobacteria infection in alveolar epithelial cells (AEC) [53]. A549 is a human type II carcinoma cell line established as a model of type II AEC [54, 55]. As such, this cell line has been extensively used to study lung infections with a range of pathogens [56–58] including mycobacteria [49, 50, 59, 60]. Besides being shielded from phagocytic cells, mycobacteria inside AEC could acquire a more infective phenotype. Indeed, it has been shown that passage of mycobacteria through alveolar epithelial cells promotes the conversion of non-virulent *M. smegmatis* to a virulent phenotype before they infect macrophages [61]. Likewise, a recent study also showed that *M. abscessus* obtained from amoeba are more infective than those growing extracellularly [62].

Understanding the survival mechanisms engaged by *M. abscessus* CRM0019 in cells other than macrophages may shed light on important aspects of its intracellular behavior that could play a role in its in vivo persistence and virulence. We investigated herein the intracellular survival of *M. abscessus* CRM0019 in comparison to reference strain *M. abscessus* ATCC 19977 in both A549 epithelial cells and macrophages, by addressing the interaction with lysosomes and reinfection capability.

## Methods

### Cell cultures

The cell lines A549 - type II alveolar epithelial cell line (DSMZ GmbH, Braunschweig, Germany) and RAW 264.7 - mouse leukemic monocyte macrophage cell line (ATCC, Manassas VA, USA) were maintained respectively in DMEM and RPMI medium (Sigma Chemical Co., St Louis, MO.), both supplemented with 10% FBS (Life Technologies, Grand Island, NY, USA), 100 μg/ml streptomycin and 100 U/ml penicillin (Sigma). Cells were grown in T75 flasks in 5% CO_2_, at 37 °C. Cultures were trypsinized upon reaching approximately 75% confluence, and concentrated by centrifugation at 360 ×*g* at 25 °C for 10 min. Cells were counted in a Neubauer chamber and viability assessed by trypan blue exclusion. Bone marrow-derived macrophages (BMDM) were obtained and cultivated as previously described [63] and used after 6-7 days of differentiation.

### Bacterial cultures

*M. abscessus* subsp. *massiliense* CRM0019 was isolated from a postsurgical wound during the 2006-2007 epidemic in Rio de Janeiro, Brazil [28]. *M. abscessus* subsp*. abscessus* smooth variant was isolated from the sputum of CF patients [27], and kindly provided by Prof. Dr. John Perry, Newcastle University. The reference strain *M. abscessus* subsp. *abscessus* ATCC 19977 was used in parallel. Both bacteria, ATCC 19977 and CRM0019, were transformed with the *Escherichia coli*-mycobacterium expression vector pMV262 containing the green fluorescent protein (GFP) gene (pMV262-*gfp*). In some experiments, *M. smegmatis* mc^2^155, wild type or transformed with the same plasmid, was used. All mycobacterial strains were grown in Middlebrook 7H9 liquid medium (Becton Dickinson, Franklin Lakes, NJ), supplemented with oleic acid, albumin, dextrose and catalase (OADC – Becton Dickinson), 0.5% glycerol and 0.05% Tween 80 at 37 °C, for 72 h. Transformed bacteria were grown in medium containing kanamycin (50 μg/ml) and sub-cultured in fresh medium for 24 h before the experiments.

### Mycobacteria-cells infection

A549 cells and macrophages (5 × 10^4^ per well) previously grown in 24-well plates in medium without antibiotics were infected simultaneously with single-cell bacterial suspensions. Accordingly, bacterial cells in exponential growth phase were pelleted, washed in PBS and suspended in DMEM. Clumps of bacteria were removed by passing through a syringe needle (25 gauge), followed by an ultrasonic water bath for 15 min and low-speed centrifugation (120 ×*g*) for 2 min. The optical densities of bacterial suspensions were adjusted to a multiplicity of infection (MOI) of 10 or 100, according to the experiment. MOI of 10 was used for macrophages and MOI of 100 for A549 cells. The experiments with *M. smegmatis* followed the same protocols as used for *M. abscessus.*

After 6 h uptake, the cells were washed with PBS to remove extracellular bacteria. Thereafter cells were incubated with DMEM containing 30 μg/ml amikacin (Sigma) to avoid extracellular bacteria growth [16]. Infected cultures were further incubated for 6, 24, 48 and 72 h at 37 °C, in 5% CO_2_.

### Colony-forming unit assay

Infected macrophages and A549 cells were washed with PBS and lysed with sterilized water. Quantitative cultures for mycobacteria were performed using 10-fold serial dilutions in PBS/0.05% Tween 80 inoculated on LB agar plates. Subsequently, 20 μl were plated in triplicate with serial dilutions. The plates were incubated at 37 °C and the colonies counted after 3 or 4 days. Results were expressed in terms of colony-forming units (CFU) per milliliter.

To verify the infectivity competence of mycobacteria in reinfection assays, mycobacteria were obtained from BMDM previously infected for 24 h. Macrophages were lysed and bacterial suspensions frozen at −20 °C with 10% glycerol. Before use in experiments, bacteria were grown in 7H9 liquid fresh medium for 72 h and prepared for cell infection as described above.

### Fluorescence microscopy

For lysosome-staining assays, A549 cells and macrophages were fixed with 3% paraformaldehyde (PFA) in PBS for 15 min at room temperature and quenched for 15 min with 50 mM glycine in PBS. Preceding immunostaining, cells were permeabilized with 0.1% saponin solution and 1% bovine serum albumin (BSA - Sigma) in PBS, for 15 min at room temperature. For A549 cells, mouse α-human and for RAW cells rabbit α-mouse LAMP-1 and α-cathepsin D antibodies (supernatants from DSHB, Iowa University, USA) were used at 1:10 dilution for 1 h. The corresponding fluorescent secondary antibodies, α-mouse-IgG for A549 cells and α-rabbit-IgG for RAW cells (AlexaFluor), were used at a dilution of 1:250 for 1 h at room temperature. Alternatively, live adherent cells were infected as described before and incubated with medium containing 200 nM LysoTracker Red DND-99 (Molecular Probes, Thermo Scientific) for 1 h at 37 °C, to evaluate phagosomal acidification. Afterwards, cells were washed with HBSS (Sigma) and coverslips were fixed with 4% PFA for 15 min.

Nuclear staining was performed using 5 nM 4,6′-diamidino-2-phenylindole (DAPI, Invitrogen, Thermo Scientific). After staining, the coverslips were mounted on slides using aqueous mounting medium (Dako Cytomation, Denmark). Samples were analyzed by epifluorescence microscopy using a Zeiss Axiovert Fluorescence microscope (Zeiss, Germany) or Leica SP5 II TS confocal microscope (Leica Microsystems, Germany) microscope. Microscopic images of fixed samples were acquired at 1024 × 1024 resolution, using a 100× (HCX PL APO 100×/1.44 CORR CS) oil immersion objective and z-stacks with steps ranging from 0.3 to 0.5 μm.

### Live cell images

Live imaging was performed by serial acquisition of images from live A549 cells pre-loaded with LysoTracker Red (200 nM) and infected with *M. abscessus* ATCC 19977 or *M. smegmatis* mc^2^155 for up to 36 h. The cells were placed in microincubators coupled to the confocal system. Images were taken every 15 min. Lasers were adjusted to no more than 5% of potency at resonant scanning mode (4000 Hz) to minimize phototoxicity. Multichamber Hi-Q4 dishes (Ibidi GmbH) were employed for experiments in which two different cell cultures, infected with different mycobacteria, were imaged in the same live imaging session. Different microscopic fields could be imaged for each condition using the multiposition tool of the confocal system.

Confocal images were analyzed with Imaris v.7.4.2 software (Bitplane AG, Andor Technology). The software allowed the construction of isosurfaces based on GFP signaling of the bacteria [64] that retrieves different morphological parameters such as bacterial volume, area and fluorescence intensity. These isosurfaces represent the region where the GFP signal of the bacteria is located in multidimensional images; they could be used to recover the fluorescence intensity of the Lysotracker Red DND-99 probe that represents the acquisition of this lysosomal probe in the bacteria-containing phagosome. Thus, the relative fluorescence intensity of LysoTracker Red detected by GFP-constructed bacterial isosurfaces is an inference of phagosomal acidification that could be measured dynamically in live cell imaging.

### Flow cytometry

A549 and RAW cells grown in 24-well plates, non-infected or infected with wild-type or GFP-expressing strains (CRM0019 or ATCC 19977) as described before were trypsinized after 6, 24, 48 and 72 h of infection and collected with 1 ml of DMEM containing 10% FBS. Cells were then centrifuged at 860 ×*g* for 10 min at room temperature, washed with PBS, and fixed with 4% PFA for 20 min at room temperature. After one additional PBS washing, the samples were resuspended in 500 μl of PBS with 10% FBS at 4 °C and immediately analyzed in a FACSCalibur flow cytometer (Becton Dickinson, San Jose, CA).

Intracellular infection was evaluated with FlowJo software (version 9.7.6, Tree Star, San Carlos, CA). For this, cells infected with wild-type *M. abscessus* or not were used as control groups. At each evaluated time, the positive fluorescent region was defined on the basis of control samples, which were GFP-negative, determining the percentage of infected cells (GFP-positive cells). The infection efficacy was determined as the fluorescence intensity of GFP-positive cells minus negative control values, and the mean fluorescence intensity (MFI) was calculated, using geometric mean value. The progression of infection by different strains over time was analyzed by comparing the fluorescence ratio, which was calculated for each time point as (GFP^+^ MFI)/(GFP^+^ MFI + GFP^−^ MFI).

### Transmission electron microscopy

Macrophages or A549 cells were grown in 75 cm^2^ flasks and infected with *M. abscessus*, clinical or reference strain, as described before. The cultures were washed with PBS and fixed with a solution containing 2.5% glutaraldehyde, 2% formaldehyde, 5 mM CaCl_2_ and 5% sucrose in 0.1 M sodium cacodylate buffer, pH 7.2, for 1 h at room temperature. Samples were post-fixed in 0.1 M sodium cacodylate buffer, pH 7.2 containing 0.8% osmium tetroxide and 2% potassium ferricyanide for 1 h, followed by dehydration with a graded series of acetone (70, 90 and 100%) and propylene oxide for 10 min each at room temperature. Afterwards, the cells were embedded in epoxy resin. Ultrathin sections (50-60 nm) were cut with a diamond knife, collected onto Butvar-coated copper grids, post-stained with 2% uranyl acetate (3 min) and lead citrate (1 min) and observed with a transmission electron microscope (Jeol 1200EX) at an accelerating voltage of 80 kV.

### Methyl-thiazolyl tetrazolium (MTT) reduction assay

Cells were seeded in 96-well plates in triplicate at a density of 1 × 10^5^ cells per well 24 h before infection. Thereafter, cells were infected for 6-72 h as described before and the viability checked with MTT (Sigma), following the manufacturer’s instructions. The plates were read at 540 nm using a plate reader (Multiskan MS, Labsystem).

### Statistical analysis

All data were recorded as mean ± SD, from 2 or 3 independent experiments, as indicated in the respective figure captions. Statistical analysis was performed using the software package Prism6^®^ (GraphPad Prism, San Diego, CA, USA). Significance was determined by t-test or two-way ANOVA, with Bonferroni posttest. Differences were considered statistically significant at *p* < 0.05.

## Results

### Comparative internalization and survival of *M. abscessus* strains

**Fig. 1:**
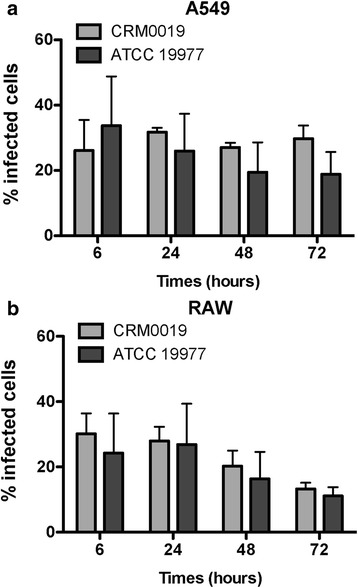
Internalization rate of *Mycobacterium abscessus* CRM0019 and *Mycobacterium abscessus* ATCC 19977. **a** and **b** Percentage of infected cells determined by flow cytometry, for both A549 (**a**) and RAW (**b**) cells. No significant difference was observed between the strains at each time point. Data represent mean +/− SD from 3 independents experiments

To evaluate *M. abscessus* infection and survival in epithelial cells (A549) and macrophages (RAW and BMDM), the clinical strain *M. abscessus* CRM0019 and the reference strain *M. abscessus* ATCC 19977 were used to infect cells for 6 to 72 h. Infected cells were fixed and analyzed by flow cytometry. The percentage of infected cells (GFP-positive cells) was determined for A549 and RAW cells (Fig. 1a, b). The internalization rate of both strains was similar in both cell lines, with approximately 30% infection after 6 h. However, for RAW cells the proportion of infected cells decreased after 72 h, to 15% in the case of both strains. Such a reduction in the percentage of infected cells is most likely due to the proliferation of uninfected cells in the same culture, as the infection itself did not induce cell death (see Additional file 1). For primary macrophages (BMDM) a similar reduction in the cell viability was observed, in both infected and uninfected cultures (see Additional file 1 C). These results are not unexpected, since primary cells are usually characterized as having a short life span.

**Fig. 2:**
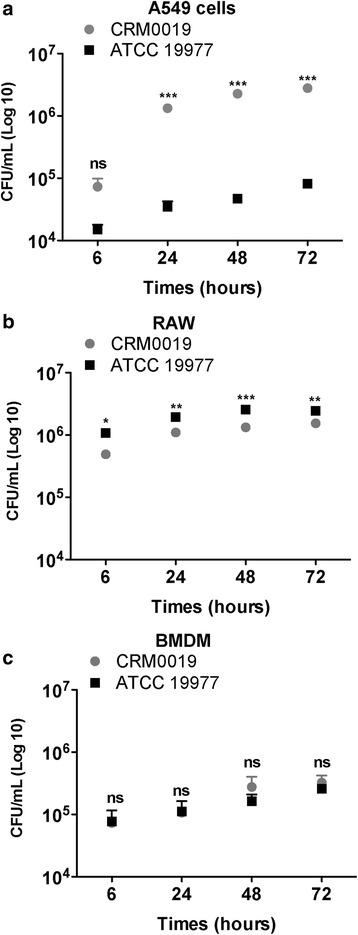
Intracellular survival of *M. abscessus* strains determined by colony forming unit (CFU) counts. *p < 0.05; **p < 0.01; ***p < 0.001; ns: not significant. Data represent mean +/− SD from 3 independents experiments

Intracellular bacterial survival was determined by CFU counting of viable bacilli over a period of 72 h. Despite the similar infection rate in A549 cells, CRM0019 showed higher survival and proliferative capability compared to the ATCC 19977 strain, resulting in sustained infection after 24 h (Fig. 2a). Similar behavior in A549 cells was observed for another clinical strain of *M. abscessus*, also with S morphotype, and recently isolated from sputum of CF patients. The efficient survival in A549 cells could also be observed in cultures without amikacin, in which, as expected, more colonies were obtained compared to the untreated culture (see Additional file 2). For both, RAW and BMDM macrophages, a similar intracellular survival of CRM0019 and ATCC 19977 strains was seen Fig. 2 b, c).

The growth rate of the two bacterial strains was determined (see Additional file 3) by calculating the ratio between CFU obtained at 6 h (Ti) and the additional time points (Tf), i.e., 24, 48 and 72 h. The clinical strain showed approximately 20-fold increase in the number of intracellular bacilli in A549 cells, while the reference strain showed an increase of approximately 5-fold. In macrophages, both strains showed the same growth rate over time, which is in line with the CFU counts obtained in Fig. 2 b, c. Despite being highly infective, *M. abscessus* did not induce host cell death during the experimental period (see Additional file 1).

**Fig. 3:**
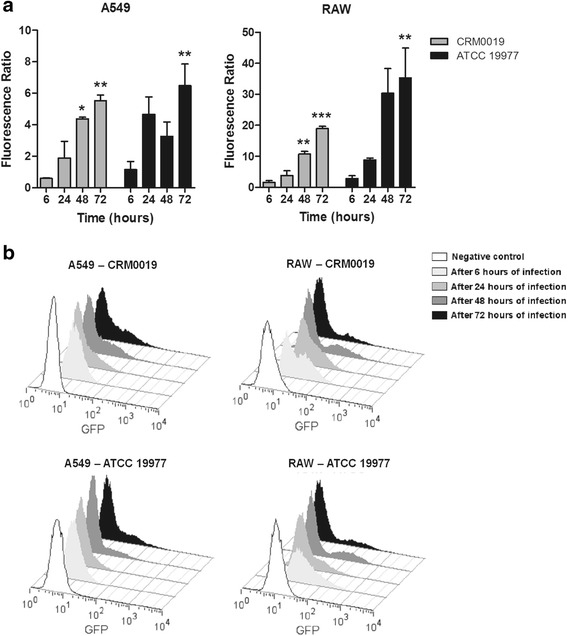
Intracellular growth of *M. abscessus* strains by flow cytometry. Internalization of strain CRM0019 or ATCC 19977 in epithelial (A549) and macrophage (RAW) cell lines was detected by flow cytometry after 6, 24, 48 and 72 h of infection. Intracellular growth was evaluated by calculating fluorescence ratio (**a**) or with histograms of GFP fluorescence (**b**). Cells infected with wild-type bacteria and uninfected cells were used as negative controls for GFP fluorescence. Statistical differences were calculated between 6 h, the first period of infection, and 24, and 72 h of infection. *p < 0.05 **p < 0.01 ***p < 0.001. Data represent mean +/− SD from 3 independents experiments

Intracellular proliferation of *M. abscessus* was also confirmed by flow cytometry (Fig. 3). The growth was evaluated by comparing cells infected with GFP-expressing bacteria either with uninfected cells or cells infected with wild-type strains. The fluorescence ratio of infected cells increased during the 72 h period of infection in A549 and RAW cells (Fig. 3 a). The increasing GFP fluorescence observed over time, visualized as a displacement of the GFP-positive population to the right in the histograms, was consistent with the increasing numbers of intracellular bacteria (Fig. 3 b).

### Phagosomal characteristics of *M. abscessus* strains in epithelial cells and macrophages

**Fig. 4:**
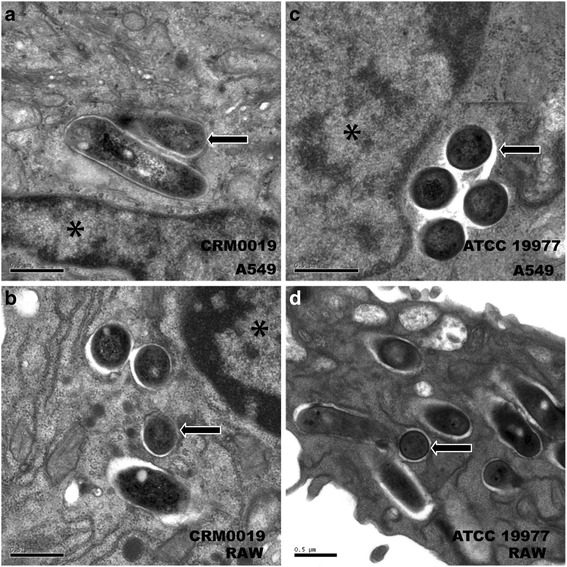
Ultrastructural characterization of *M. abscessus* phagosomes after 24 h of infection. **a**, **b** *M. abscessus* CRM019 in A549 (**a**) or RAW (**b**) cells; (**c**, **d**) *M. abscessus* ATCC 19977 in A549 (**c**) or RAW (**d**) cells. Asterisks: host cell nuclei; arrow: intravacuolar bacteria; Bar: 0.5 μm

Ultrastructural analyses and phagosome acidification studies were carried out to characterize phagosomes containing *M. abscessus* as well as host cell-lysosomal system interaction. After 24 h of infection, *M. abscessus* ATCC 19977 and *M. abscessus* CRM0019 were localized inside vacuoles in A549 cells and macrophages (Fig. 4). Representative TEM pictures show the characteristics of each strain, either in A549 or RAW cells. A549 phagosomes containing the clinical strain showed their membrane closely apposed to the mycobacterial cell surface, characteristic for R strains (Fig. 4 a). In RAW cells however, CRM0019 was also located in single phagosomes, but the phagosomal membrane was separated from the bacteria cell wall (Fig. 4 b). For the reference strain ATCC 19977, phagosomes showed a “loose” apposed membrane in both cell types (Fig. 4 c, d), characteristic of S variant strains. Moreover, most of the phagosomes here contained several bacteria.

**Fig. 5:**
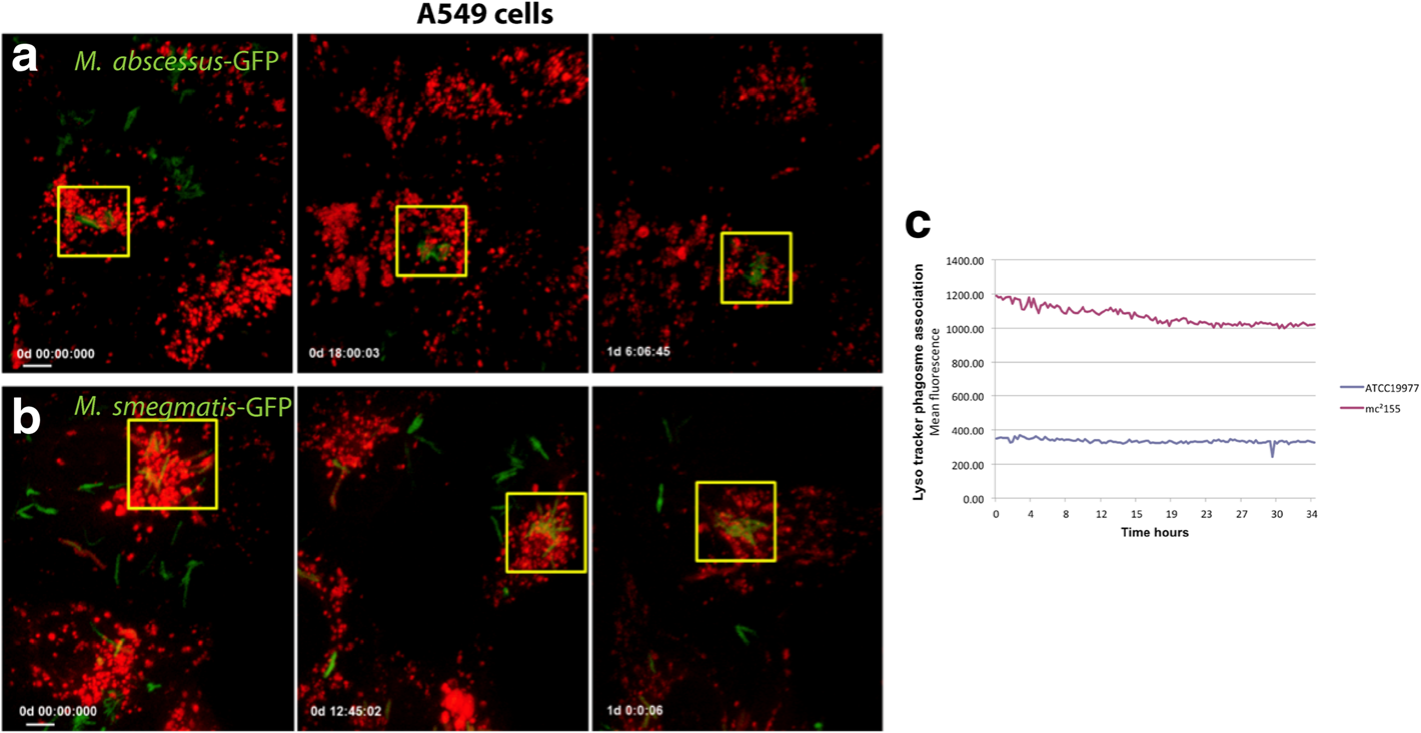
Live cell imaging of infected A549 cells pre-loaded with LysoTracker Red. **a** *M. abscessus* ATCC 19977 and (**b**) *M. smegmatis* infection in A549 cells for 36 h. Time of image acquisition is depicted as days:hours:minutes:seconds (d:hh:mm:ss) and image acquisition started after 2 h of infection. **c** Mean fluorescence intensity of LysoTracker Red quantified from square regions in A and B. Total of 5 cells containing phagosomes were analyzed per condition. Bar: 10 μm

**Fig. 6:**
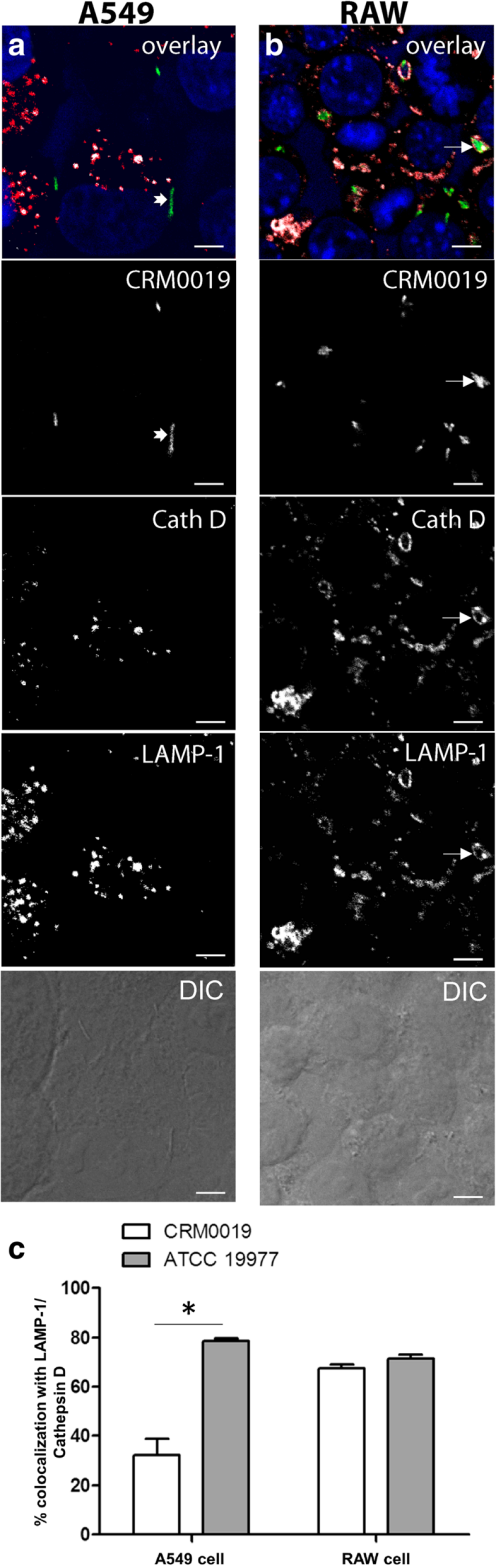
Colocalization of lysosomal proteins in *M. abscessus* CRM0019 phagosomes. **a**-**f** Z-stack images were obtained from A549 (**a**) and RAW (**b**) cells infected with *M. abscessus* CRM0019 for 24 h. **a** and **b**: Colocalization of mycobacteria-GFP, LAMP-1 and Cathepsin D, followed by the respective separated channels of CRM0019-GFP, Cathepsin D, LAMP-1 and transmitted light (DIC). **c** Quantification of *M. abscessus* CRM0019 and *M. abscessus* ATCC 19977 phagosomes simultaneously positive for LAMP-1 and cathepsin D in A549 and RAW cells. *P < 0.05. 200 phagosomes were counted per condition. Bar: 10 μm

Phagosome maturation was evaluated in infected A549 and RAW cells previously loaded with the lysosomal marker LysoTracker Red. *M. smegmatis* mc^2^155 was used in parallel as a control mycobacterium that allows lysosome fusion [60, 65]. Live cell images were taken to follow mycobacterium phagosome acidification in LysoTracker Red pre-loaded A549 cells (Fig. 5). *M. smegmatis* phagosomes were much more acidified than *M. abscessus* phagosomes (Fig. 5 a, b). The sequence of images of *M. abscessus* phagosomes (Fig. 5 a) showed bacteria-GFP surrounded by LysoTracker Red-positive vesicles without a clear colocalization of phagosomes and mycobacteria. Similar results were observed for CRM0019 after 48 h infection in A549 cells, in which bacteria showed slight colocalization with acidic vesicles (see Additional file 4). With *M. smegmatis* there was evident colocalization of the mycobacteria (Fig. 5 b), and LysoTracker Red was observed in some recorded phagosomes, particularly in the first hours. Square regions of the multidimensional images (in yellow) were selected to measure LysoTracker Red intensity associated with the bacteria (Fig. 5 c). The mean LysoTracker Red fluorescence intensity in regions where mycobacteria were located was retrieved in arbitrary units and plotted in relation to the acquisition time points (in hours). *M. abscessus* ATCC 19977 phagosomes in A549 cells showed lower LysoTracker Red fluorescence intensity compared to the intensity produced by *M. smegmatis* infection (Fig. 5 c). To closely follow phagosomal maturation, A549 and RAW cells infected with the two *M. abscessus* strains were simultaneously immunolabeled with LAMP-1 and cathepsin D. Z-stack images of *M. abscessus*-infected A549 and RAW cells showed the colocalization of cathepsin D and LAMP-1 in the CRM0019 phagosomes (Fig. 6 a, b) as well as in the ATCC 19977 phagosomes (see Additional file 5). Almost 80% of the vacuoles containing *M. abscessus* ATCC 19977 were positive for both lysosomal markers, in either A549 or RAW cells (Fig. 6 c). However, for the clinical strain CRM0019 only 30% of the vacuoles were positive for LAMP-1 and cathepsin D in A549 cells. A different result was obtained with RAW cells, in which the maturation of *M. abscessus* CRM0019 phagosomes was similar to that observed for *M. abscessus* ATCC 19977, where there was almost 80% lysosomal protein colocalization.

### Reinfection of *M. abscessus* strains obtained from macrophages

**Fig. 7:**
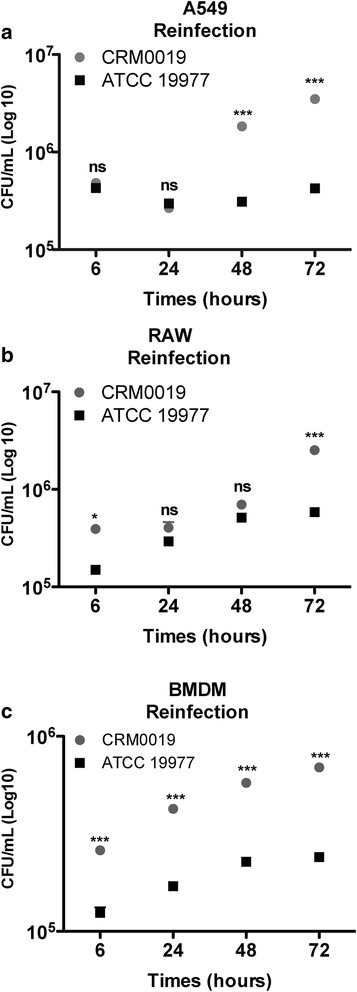
Intracellular survival of *M. abscessus* CRM0019 and *M. abscessus* ATCC 19977 after cell-reinfection. **a** A549, **b** RAW and **c** BMDM were infected with both strains, previously obtained from BMDM. In parallel, intracellular growth was determined by CFU counts. Data represent mean ± SD from 3 independent experiments. *p < 0.05; ***p < 0.001; ns: not significant

Because of the evidence that mycobacteria become more infective after the first intracellular infection [61, 62], we evaluated whether this holds for *M. abscessus* obtained from macrophages. The aforementioned results showed that *M. abscessus* were highly internalized by macrophages and ended up in a mature lysosomal compartment without, however, evidence of intracellular death (Figs. 2 and 6). Mycobacteria obtained from BMDM previously infected for 24 h were used in reinfection assays of A549 cells or new macrophages (RAW and BMDM) (Fig. 7). The clinical strain showed increased growth in A549 cells compared to ATCC 19977 (Fig. 7), in which higher CFU counts were obtained after 48 and 72 h (Fig. 7 a). In RAW cells, this was observed only after 72 h (Fig. 7 b); in the case of BMDM, the number of colonies obtained with CRM0019 was slightly higher compared to the reference strain (Fig. 7 c). CRM0019 showed a higher growth rate in A549 cells compared to the reference strain, mainly after 48 and 72 h (see Additional file 6), with a nearly 10-fold increase. For both macrophage lines, the results were similar to those observed in the first infection, except for RAW cells infected with CRM0019 at 72 h (see Additional file 3). Mycobacterial growth rate was also determined for *M. smegmatis,* which showed no colonies after reinfection of all cell types (data not shown).

## Discussion

*M. abscessus* subsp. *massiliense* CRM0019 survives in macrophages and also subverts the immune response in vivo, which may contribute to its increased persistence [32]. Besides the ability to produce biofilms, limiting the efficacy of treatment, intracellular growth may also contribute to the successful survival of *M. abscessus*. In culture this bacteria exhibits two different colony morphologies referred to as rough (R) and smooth (S) morphotypes [21]. Despite the R morphotype being considered more virulent, S variants have been identified as the dominant morphotype obtained from CF patients [23]. Likewise, CRM0019 obtained during an epidemic of postsurgical infections, displays an S morphotype (“personal communication” by Dr. Rafael Duarte), and is characterized by an aggressive and invasive behavior in vivo [30].

For several decades, observations from in vivo histological samples of patients or animal models have shown that macrophages are the predominant host cells for mycobacteria. However, bacilli may be released from macrophages and infect cells other than macrophages thus establishing a stable or chronic infection. A recent study using a human ex vivo lung model showed that besides immune cells, Type II pneumocytes were also infected with *M. tuberculosis*, *M. avium* and *M. abscessus* [53]. For the isolate CRM0019 we know from previous studies that this strain is highly virulent in vitro and causes progressive lung infections in mice [32].

Based on these observations we investigated the intracellular survival of the outbreak strain *M. abscessus* CRM0019, focusing on its interaction with the primary host defense system - lysosomal digestion, and its reinfection capability.

### Uptake of *M. abscessus* CRM0019 in A549 cells and macrophages

There is considerable evidence towards the role of epithelial cells as a replicative niche for *Mycobacterium* survival [47–52, 54]. Herein, we showed similar internalization rates for the clinical strain *M. abscessus* CRM0019 as for the reference strain ATCC 19977 in A549 cells. As expected, both strains were internalized by macrophages, and the survival of *M. abscessus* was in line with previously published data [16, 35, 66]. It has been shown that S and R mycobacteria morphotypes have distinct uptake efficacies in macrophages, most likely due to the intrinsic clumping behavior of R strains [35]. Despite our extensive procedures to obtain single bacteria preparations, we also noticed a higher clumping tendency of the clinical strain, which is atypical for S morphotypes. Even so, this did not interfere with the uptake efficacy of CRM0019, either in A549 or RAW cells.

It is well known that the respiratory epithelium plays an important role in host innate immune responses to bacterial pathogens, through binding of the bacteria to TLR (toll like receptors). *M. abscessus* variant lacking GPL trigger immune responses in A549 cells through TLR, contrasting to the S variant that is not recognized by TLR2 [33]. Thus, we cannot exclude that CRM0019 can subvert innate immune responses triggered by TLR binding. Such a strategy should therefore be further investigated for *M. abscessus* CRM0019 strain.

### Intracellular survival and intraphagosomal characteristics of *M. abscessus* CRM0019 strain

In contrast to the similar internalization rate of CRM0019 and ATCC 19977 in A549 cells, the intracellular survival of CRM0019 was higher than the reference strain as evidenced by CFU counts and flow cytometry data. As amikacin was maintained during the entire experiment, we believe that extracellular growth is quite unlikely to contribute to the increased CFU number, contrasting to what we observed in infected cultures without amikacin (see Additional file 2). Such higher survival of CRM0019 was unexpected and indeed more correlated to R variant behavior [16]. This is contradictory since the CRM0019 strain has S morphotype upon isolation.

It is possible that the low CFU number obtained from ATCC 19977 infection in A549 cells is due to a killing activity against this strain. Since ATCC 19977 was isolated in 1953, we cannot exclude a possible attenuation after several passages in a laboratory setting. Another reference strain, *M. massiliense* CIP 108297 – isolated in 2004 from the sputum of a patient with pneumonia - also showed lower survival capability compared to the clinical isolate CRM0019 [32]. Nevertheless we believe that the difference between ATCC 19997 and CRM0019 survival might be related to the clinical significance of CRM0019 rather than only to the attenuation of the reference strain. Our hypothesis is supported by the results we obtained with another clinical strain (*M. abscessus* subsp. *abscessus*), recently isolated from CF patients, which showed higher survival in A549 cells, suggesting therefore that wild strains share the potential to cause disease.

Epithelial cells may function as a protective niche for *M. abscessus* survival, and there is existing evidence to support this hypothesis. The first piece of evidence is from recent transcriptional data showing upregulation of genes in Type II epithelial cells related to the active proliferation of *M. tuberculosis* [52]. Moreover, *M. abscessus* isolated from human endophthalmitis cases exhibited increased intracellular survival in endothelial cells and higher levels of nitric oxide production compared to *M. smegmatis* or *M. tuberculosis* [36]. Thus, the evidence of higher proliferation of pathogenic mycobacteria in cells other than macrophages supports our results, in which CRM0019 shows a more infective profile than the reference strain in A549 cells.

The success of pathogenic mycobacteria depends on their ability to survive inside vacuoles and to prevent phagosome maturation [67–69] and for CRM0019 strain this may be no different. Our findings show that in both RAW or BMDM macrophages clinical and reference strains of *M. abscessus* were localized in acidified compartments while maintaining proliferative capacity. This is in contrast to the results obtained with *M. smegmatis*, in which the bacilli were, as expected, eliminated in macrophages. Regardless of the acidic nature of *M. abscessus* phagosomes, no bacterial death was observed up to 72 h of infection, noted by the constant number of viable colonies (CFU). Such behavior is in line with other findings confirming intracellular survival of *M. abscessus* in macrophages [16, 35, 66, 70]. Indeed, the successful survival of pathogenic mycobacteria in the potentially hostile environment of macrophages has been extensively reported for pathogenic mycobacterium like *M. tuberculosis*, *M. bovis* or *M. bovis* BCG [39, 69, 71–73]. Such persistent behavior results in a sustained intracellular survival for several days [71, 73], being also facilitated by the emergence of multidrug-resistant strains [74].

Macrophages represent the first line of cellular defense against pathogens, and it is well known that *M. tuberculosis* is able to subvert the defense machinery of these cells, which is characterized by production of toxic oxygen and nitrogen radicals, as well as phagosome maturation. Lysosome fusion is the end point to degrade internalized microbes. Intracellular survival of *M. abscessus* CRM0019 not only in macrophages but also in A549 cells, suggests that this strain has additional strategies to counteract the intracellular defense machinery. Our results showed that one difference is the acquisition of lysosomal proteins by CRM0019, which was not as significant in RAW as in A549 cells.

Ultrastructural analysis of *M. abscessus* CRM0019 and ATCC 19977-containing phagosomes showed that in RAW macrophages, both bacterial strains reside within phagosomes, usually consisting of a single bacterium. Such a characteristic is also supported by recent work from Roux and collaborators [35]. In their report, the S variant exhibited a thick GPL-rich cell wall, apposed to the phagosomal membrane, which is in line with our findings. Nevertheless, in A549 cells, the phagosomal membrane was tightly adherent to the CRM0019 cell wall, a characteristic usually observed in R variants [16, 35]. Conversely, the ATCC 19977 strain had the phagosomal membrane loosely associated to its cell wall, a finding that supports characteristics of S variant, previously described by other groups [16, 35]. Observations by the de Chastellier group suggested that pathogenic mycobacteria have a close apposition of the phagosome membrane, and that such an arrangement was crucial to prevent phagosome maturation [75]. CRM0019 also showed a close membrane apposition to the phagosome in A549 cells, explaining the prevention in acquisition of lysosomal proteins in the phagosomes. Unexpectedly, these characteristics could not be observed in macrophages.

It has been widely described that S and R variants have different behavior with regard to their in vivo interactions as well as with eukaryotic cells. *M. abscessus* bacteria with S phenotype avoid immune responses from epithelial cells, contrasting to bacteria with R phenotype [33]. The S variant shows indeed an attenuated ability to infect murine hosts and to persist in human monocytes [16]. From our results, the infection of CRM0019 in A549 cells had some peculiar characteristics generally observed for the R morphotype, for instance the higher survival. Nonetheless, this strain showed S morphotype upon isolation. A spontaneous transition from S to an R phenotype by *M. abscessus* CRM0019 cannot be excluded, since it could contribute to the establishment of an aggressive and persistent infection. Some groups have already shown the switch between S to R variants in CF strains [25, 76].

### Reinfection of *M. abscessus* strains obtained from macrophages

Following respiratory infection, alveolar macrophages are essential to eliminate inhaled microbes. Nevertheless, pathogens can overcome this primary immune defense and spread the infection, which seems to be the case for *M. abscessus* infection. Infected macrophages are also able to migrate through epithelial and endothelial barriers, which may contribute to extrapulmonary infections [77]. Within this scenario, we examined the infective competence of *M. abscessus* after first infection in macrophages, in which the bacilli were in a stationary growth phase and harbored within mature phagosomes (Figs. 2 and 6). Although second infection occurred to a lesser degree when compared to initial infection, only the clinical strain CRM0019 grew in A549 cells and macrophages in a second infection, which may explain the long persistence of the observed in vivo infection [32]. Evidence suggests that intracellular passage through epithelial cells changes the mycobacterial phenotype to a more pathogenic one during infection of macrophages, epithelial or endothelial cells [61, 77, 78]. In our study, mycobacteria obtained from macrophages were not more infective but were still able to survive in either macrophages or alveolar epithelial cells. Whether the S morphotype of CRM0019 strain spontaneously switches to an R morphotype after a first infection in macrophages is an open question that should be further investigated. We believe that the release of bacteria by macrophages may contribute to subsequent infections and their persistence in the host. Recently, it was elegantly shown in a zebrafish model that macrophages harboring *M. abscessus* undergo apoptosis and release bacteria into the extracellular medium, which can be pathogenic and induce lethal infections [79]. Future work addressing genomic and transcriptomic changes in *M. abscessus,* obtained from a first infection in macrophages or in alveolar epithelial cells, would help to understand the adaptation of this bacteria to the intracellular environment.

## Conclusion

In conclusion, this work demonstrated that *M. abscessus* CRM0019 survives in alveolar epithelial cells mainly due to the inhibition of phagosome maturation. Macrophages containing these bacteria are not able to kill them despite the mature characteristics of their phagosomes. Finally, *M. abscessus* CRM0019 recovered from macrophages are able to establish a new intracellular infection, which may explain the aggressive profile of this strain.

## Additional files

Additional file 1: Cell viability after *M. abscessus* infection. (A) A549, (B) RAW and (C) BMDM were infected for 6-72 h with *M. abscessus* CRM0019. Infection did not induce evident cytotoxicity during this time. Data represent mean ± SD from 2 independent experiments. No statistical difference was observed for each time point. (TIFF 225 kb)

Additional file 2: Intracellular survival of *M. abscessus* subsp. *abscessus* smooth (MABS), isolated from CF patients. A549 cells infected with *M. abscessus* were incubated with or without amikacin, for 6-72 h. Significant differences between treated and untreated are indicated at each time point. Data represent mean ± SD from 2 independent experiments. *p < 0.05, ***p < 0.001. (TIFF 148 kb)

Additional file 3: Growth rate of *M. abscessus* strains. The CFU obtained in Fig. 2c was used to calculate the growth rate of *M. abscessus* CRM0019 or *M. abscessus* ATCC 19977 in (A) A549, (B) RAW or (C) BMDM cells. Tf = 24, 48 or 72 h and Ti = 6 h. Data represent mean ± SD from 3 independent experiments. ***p < 0.001; ns: not significant. (TIFF 314 kb)

Additional file 4: *M. abscessus* CRM0019 phagosome acidification in A549 cells after 48 h infection. (A) overlay of CRM0019-GFP and Lyso tracker red; (B) CRM0019; (C) Lyso tracker red. Arrow: intracellular bacteria loosely associated with acidic vesicles. Bar: 10 μm. (TIFF 14938 kb)

Additional file 5: Colocalization of lysosomal proteins in *M. abscessus* ATCC 19977 phagosomes. (A-F) Z-stack images were obtained from RAW infected for 24 h. (A) Mycobacteria-GFP; (B) LAMP-1: (C) Cathepsin D; (D) Colocalization of A, B and C; (E) Transmitted light; (F) Colocalization of GFP, LAMP-1 and DAPI. Bar: 10 μm. (TIFF 27030 kb)

Additional file 6: Growth rate of *M. abscessus* CRM0019 and *M. abscessus* ATCC 19977 after reinfection. (A) A549, (B) RAW or (C) BMDM cells. Growth rate was determined by the ratio Tf/Ti, in which Tf = 24, 48 or 72 h and Ti = 6 h. ***p < 0.001; ns: not significant. (TIFF 365 kb)

## Abbreviations

AEC: Alveolar epithelial cell
BMDM: Bone marrow-derived macrophages
CFU: Colony-forming units
GFP: Green fluorescent protein
GPLs: Glycopeptidolipids
MTT: Methyl-thiazolyl tetrazolium
NTM: Nontuberculous mycobacterium
R: Rough
RGM: Rapid-growing mycobacteria
S: Smooth
TLR: Toll like receptors
