# Supplementary figures and images for: Increased survival and proliferation of the epidemic strain Mycobacterium abscessus subsp. massiliense CRM0019 in alveolar epithelial cells
Source: BMC Microbiol. 2017 Sep 13;17:195. doi: 10.1186/s12866-017-1102-7 (PMC5598063; doi:10.1186/s12866-017-1102-7)

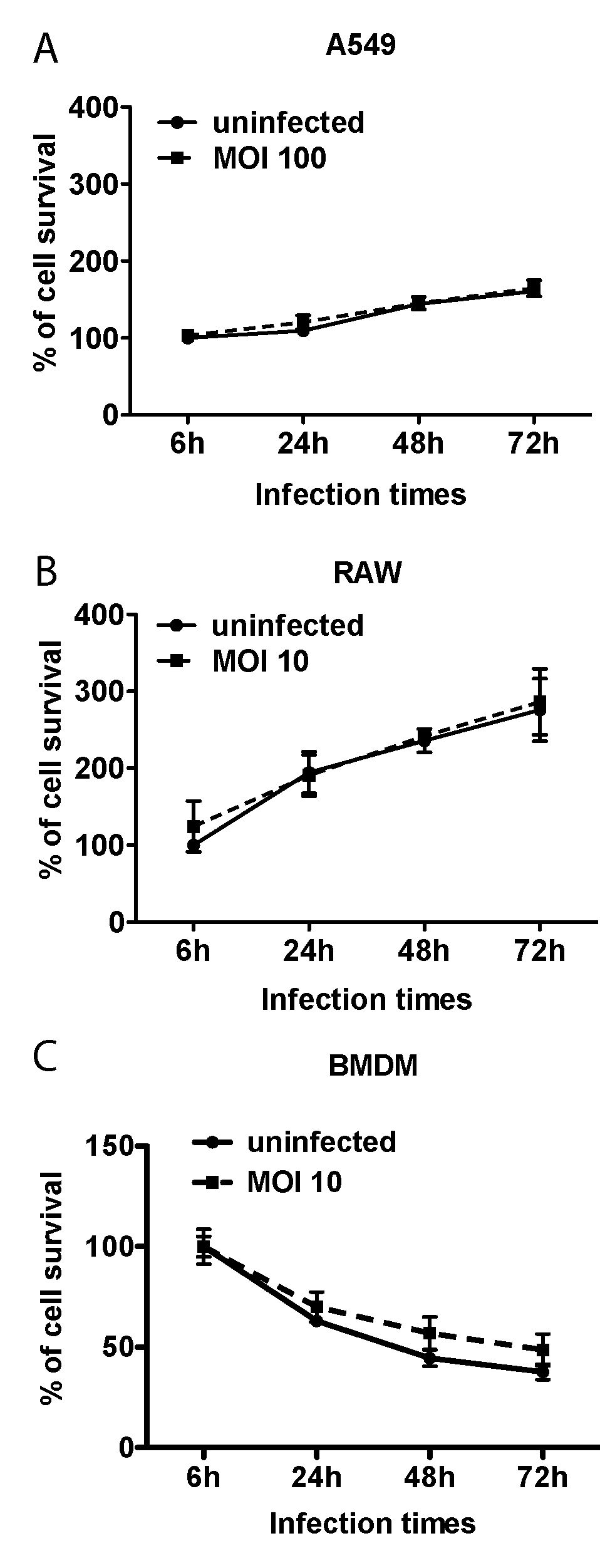

Supplement: Supplementary file 1 — Cell viability after M. abscessus infection. (A) A549, (B) RAW and (C) BMDM were infected for 6-72 h with M. abscessus CRM0019. Infection did not induce evident cytotoxicity during this time. Data represent mean ± SD from 2 independent experiments. No statistical difference was observed for each time point. (TIFF 225 kb) [file 12866_2017_1102_MOESM1_ESM.tif]

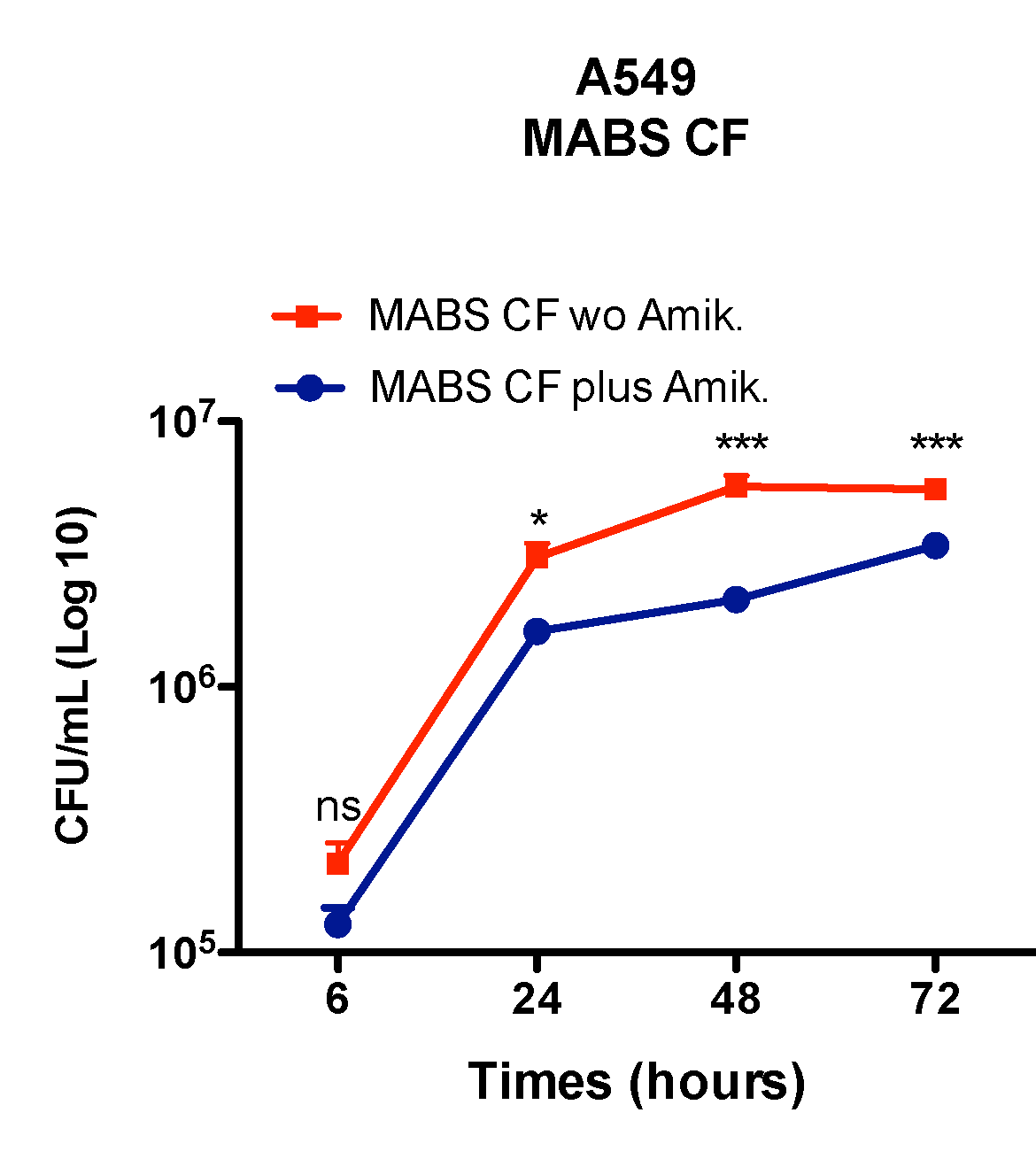

Supplement: Supplementary file 2 — Intracellular survival of M. abscessus subsp. abscessus smooth (MABS), isolated from CF patients. A549 cells infected with M. abscessus were incubated with or without amikacin, for 6-72 h. Significant differences between treated and untreated are indicated at each time point. Data represent mean ± SD from 2 independent experiments. *p < 0.05, ***p < 0.001. (TIFF 148 kb) [file 12866_2017_1102_MOESM2_ESM.tif]

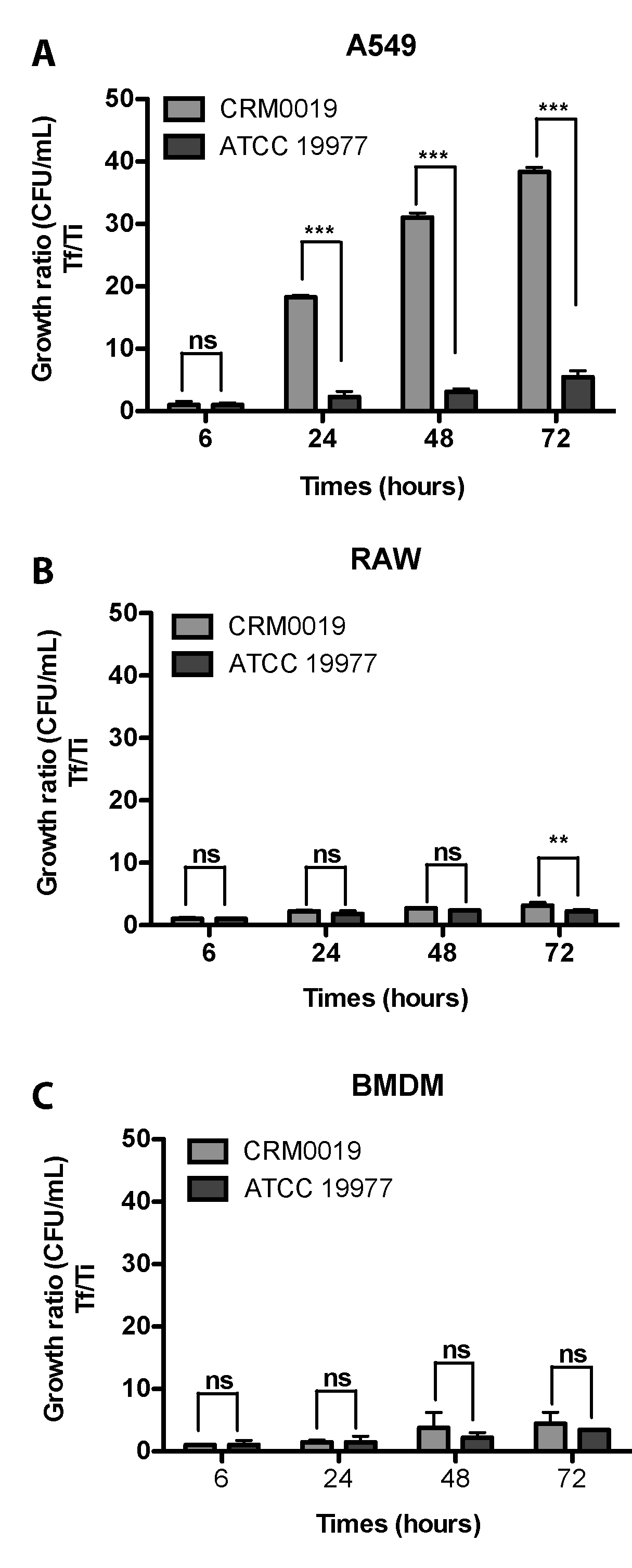

Supplement: Supplementary file 3 — Growth rate of M. abscessus strains. The CFU obtained in Fig. 2c was used to calculate the growth rate of M. abscessus CRM0019 or M. abscessus ATCC 19977 in (A) A549, (B) RAW or (C) BMDM cells. Tf = 24, 48 or 72 h and Ti = 6 h. Data represent mean ± SD from 3 independent experiments. ***p < 0.001; ns: not significant. (TIFF 314 kb) [file 12866_2017_1102_MOESM3_ESM.tif]

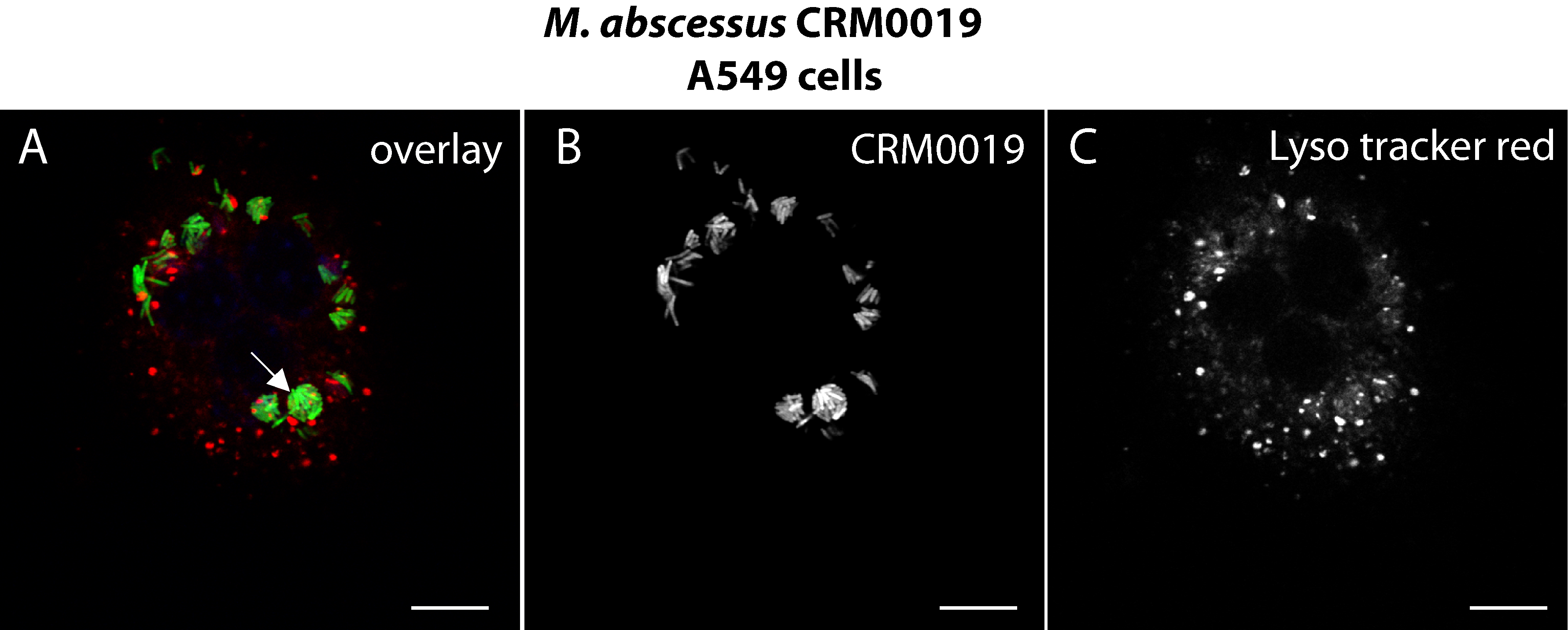

Supplement: Supplementary file 4 — M. abscessus CRM0019 phagosome acidification in A549 cells after 48 h infection. (A) overlay of CRM0019-GFP and Lyso tracker red; (B) CRM0019; (C) Lyso tracker red. Arrow: intracellular bacteria loosely associated with acidic vesicles. Bar: 10 μm. (TIFF 14938 kb) [file 12866_2017_1102_MOESM4_ESM.tif]

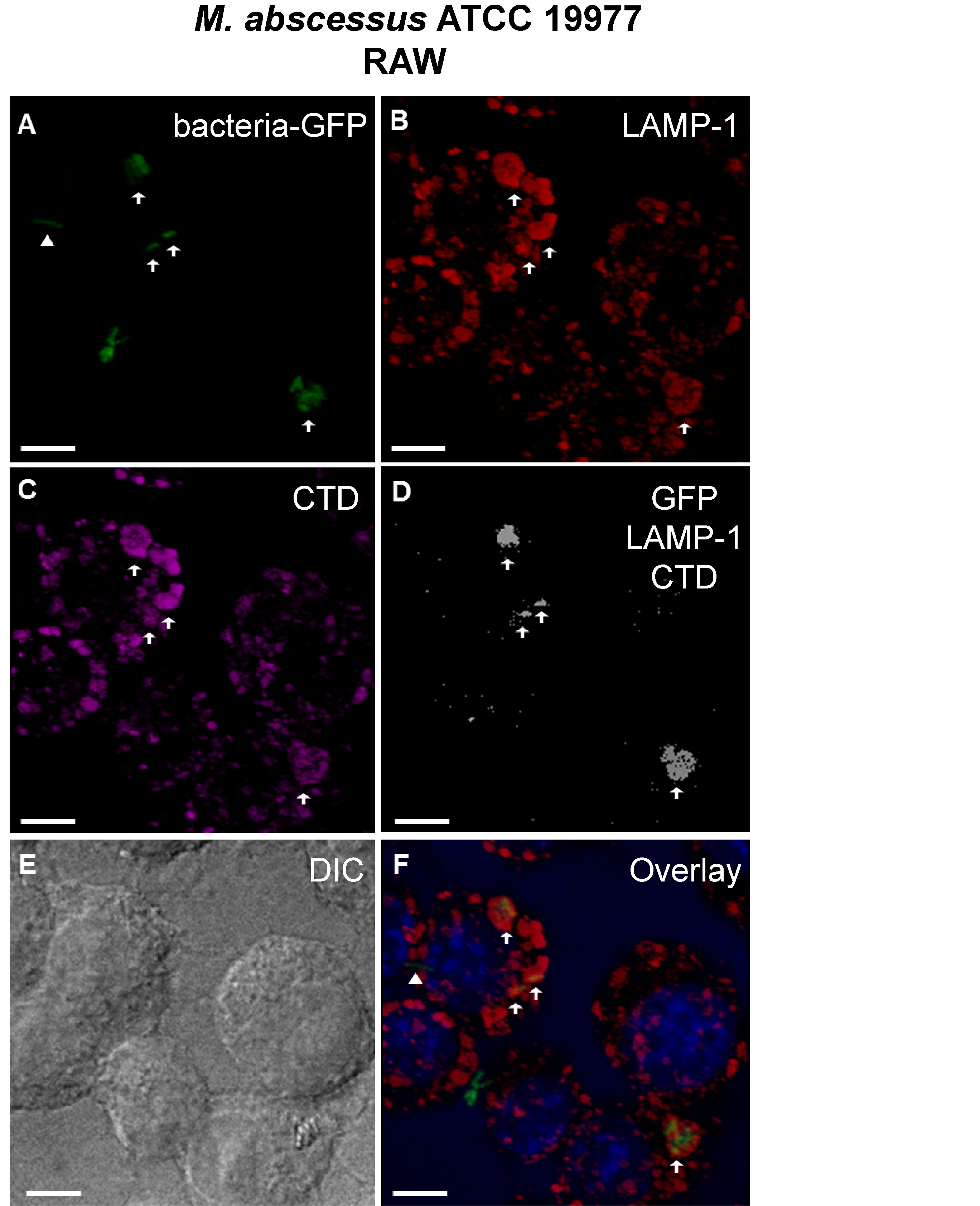

Supplement: Supplementary file 5 — Colocalization of lysosomal proteins in M. abscessus ATCC 19977 phagosomes. (A-F) Z-stack images were obtained from RAW infected for 24 h. (A) Mycobacteria-GFP; (B) LAMP-1: (C) Cathepsin D; (D) Colocalization of A, B and C; (E) Transmitted light; (F) Colocalization of GFP, LAMP-1 and DAPI. Bar: 10 μm. (TIFF 27030 kb) [file 12866_2017_1102_MOESM5_ESM.tif]

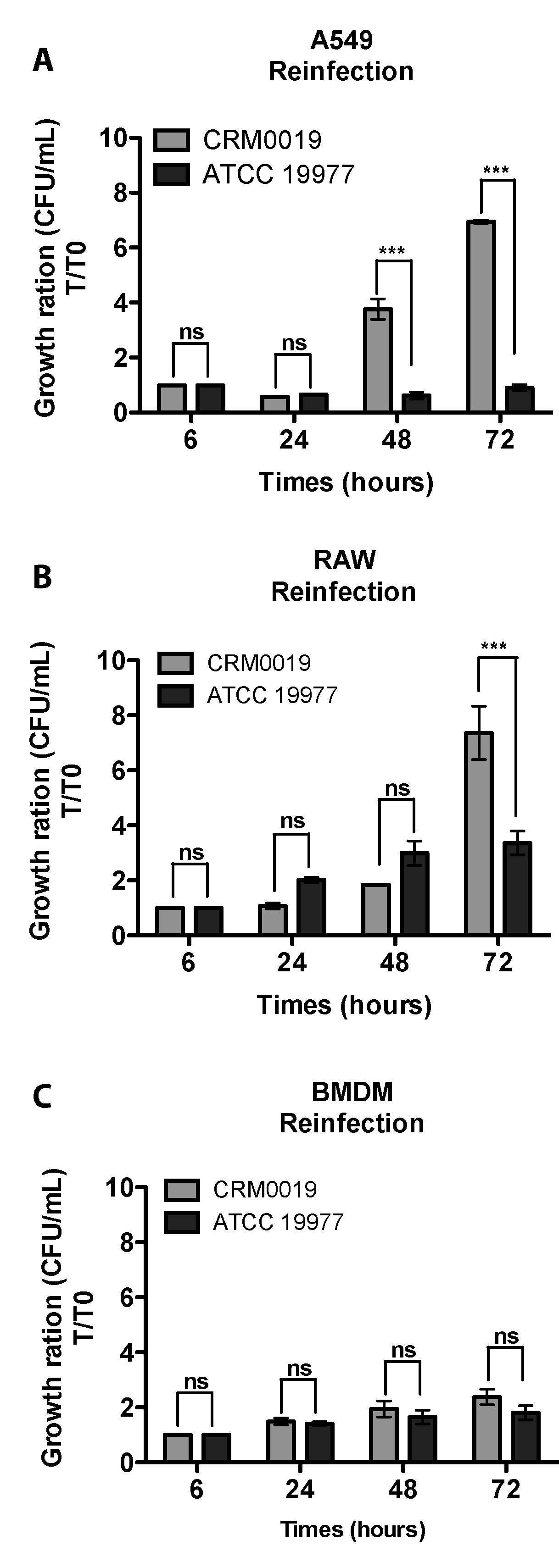

Supplement: Supplementary file 6 — Growth rate of M. abscessus CRM0019 and M. abscessus ATCC 19977 after reinfection. (A) A549, (B) RAW or (C) BMDM cells. Growth rate was determined by the ratio Tf/Ti, in which Tf = 24, 48 or 72 h and Ti = 6 h. ***p < 0.001; ns: not significant. (TIFF 365 kb) [file 12866_2017_1102_MOESM6_ESM.tif]
